# Delirium in Critical Illness Patients and the Potential Role of Thiamine Therapy in Prevention and Treatment: Findings from a Scoping Review with Implications for Evidence-Based Practice

**DOI:** 10.3390/ijerph18168809

**Published:** 2021-08-20

**Authors:** Sandra Lange, Wioletta Mędrzycka-Dąbrowska, Adriano Friganovic, Ber Oomen, Sabina Krupa

**Affiliations:** 1 Department of Anesthesiology and Intensive Care, Hospitals Tczewskie SA, 83-110 Tczew, Poland; langa94@gumed.edu.pl; 2 Department of Anesthesiology Nursing & Intensive Care, Faculty of Health Sciences, Medical University of Gdansk, 80-211 Gdańsk, Poland; 3 Department of Anesthesiology and Intensive Medicine, University Hospital Centre Zagreb, 10000 Zagreb, Croatia; adriano@hdmsarist.hr; 4 Department of Nursing, University of Applied Health Sciences, 10000 Zagreb, Croatia; 5 European Specialist Nurses Organization (ESNO), 6821HR Arnhem, The Netherlands; secretariat@esno.org; 6 Institute of Health Sciences, College of Medical Sciences, University of Rzeszow, 35-310 Rzeszow, Poland; sabinakrupa@o2.pl

**Keywords:** delirium, thiamine, critical illness, ICU

## Abstract

Introduction: Thiamine is a water-soluble vitamin and is necessary for energy metabolism. Critically ill patients are at particular risk of developing thiamine deficiency and related complications. One of the complications that can occur is delirium. Delirium is a disorder that affects the body’s response to treatment, length of stay in the ward, mortality, long-term cognitive impairment, and significantly increases treatment costs. In addition, studies show that delirium medication is more effective in preventing than in treating delirium. Given its low cost, availability, and minimal risk of side effects, thiamine supplementation could prove to be a relevant strategy in the prevention and treatment of delirium. Methods: PubMed, Cochrane Library, Ovid, and ClinicalTrials.gov databases were searched using relevant keywords that focus on the use of thiamine to prevent or treat delirium in critically ill patients. Results: Seven articles were included in the analysis. Conclusion: The small number of studies and considerable heterogeneity prevent conclusions supporting the use of thiamine as an adjuvant in the prevention and treatment of delirium among critically ill patients. There is a need for high-quality, large-scale randomized clinical trials to confirm the beneficial effects of thiamine in the prevention and treatment of delirium.

## 1. Introduction

Recently, there has been an increased interest in vitamins and their use in therapy among critically ill patients [1]. The effect of thiamine supplementation has been studied in various disease states, including sepsis and heart failure [2,3,4]. It is estimated that 11–70% of patients in the ICU (intensive care unit) are thiamine deficient (excluding thiamine deficiency in the course of alcoholic disease) [5]. Symptoms of deficiency are non-specific and often not diagnosed [6]. Thiamine deficiency may cause complications in patients hospitalized in ICU, such as delirium, gastrointestinal dysfunction, heart failure, critical neuropathy, or unexplained lactate acidosis [7]. Thiamine supports normal neuronal activity and is essential for cellular energy production, glycolysis, and oxidative metabolism [1,8]. Delirium affects up to 80% of ICU patients and is a symptom of acute brain dysfunction [9]. Prevention, removal of modifiable risk factors, and early recognition of delirium symptoms are effective non-pharmacological interventions for delirium prevention. Evidence-based medicine (EBM) analysis results to date have not identified an effective pharmacological strategy. Thus, there is a need to develop a safe and effective pharmacological strategy for the prevention and treatment of delirium [10]. Beneficial effects on delirium prevention have been shown with melatonin and its antagonists, but clinical data are inconclusive [10]. In our work, we would like to focus on a review of the literature and clinical trials that examine the effect of thiamine administration on delirium prevention and treatment.

### 1.1. Thiamine and Delirium—Pathophysiology

Thiamine is a water-soluble vitamin not produced by the body, meaning that it must be supplied with food. It is one of the essential B vitamins involved in energy metabolism [11]. Thiamine is stored in the body in minimal amounts for up to 18 days [12]. The recommended daily intake of thiamine is 1.1 mg for women and 1.2 mg for men [13]. In addition, there are medical states in which patients are particularly vulnerable to thiamine deficiency and consequently to thiamine-related complications [5].

Delirium is a neuropsychiatric disorder manifested by acute onset, cognitive impairment, disorientation, and impaired consciousness [13,14]. The incidence of this complication is quite high and may reach up to 80% [9]. Moreover, delirium adversely affects the patient’s treatment process by prolonged ICU hospitalization, longer durations of mechanical ventilation, higher mortality, and higher treatment costs [9,15,16,17,18].

Recently, delirium, its treatment and prevention have been of interest to many researchers [19,20]. Several studies have associated the occurrence of delirium and hallucinations with thiamine deficiency [8]. Enzymes that need vitamin B1 as a cofactor are present in all body cells. Converted into its active form, thiamine pyrophosphate, vitamin B1 is an essential cofactor in two carbohydrate metabolic pathways: the Krebs cycle and the pentose phosphate pathway (PPP) [8]. In the Krebs cycle, thiamine is an essential cofactor for two major enzymes, the pyruvate dehydrogenase complex (PDH) and α-ketoglutarate dehydrogenase (α-KGDH) [21,22]. The reduction of ATP production in the brain results in increased production of toxic dopamine metabolites and inhibition of the enzyme that synthesises and breaks down dopamine in the subfrontal cortex. Increased dopamine levels can lead to delirium, hallucinations [7]. Another association with increased cognitive impairment and delirium is low levels of acetylcholine, which is linked to PDH function [22]. PDH is involved in the production of the neurotransmitter acetylcholine and in the synthesis of myelin [21]. The Krebs cycle and α-KGDH are also involved in maintaining levels of gamma-aminobutyric acid (GABA), glutamate, and aspartate [23]. A decrease in levels of the inhibitory neurotransmitter GABA and an increase in glutamine leads to neuronal excitation, resulting in delirium [7,24]. The PPP is an alternative to glycolysis [8]. In this pathway, thiamine is an essential cofactor for the enzyme transketolase, which modifies the glucose-6-phosphate moiety to the compound rubose-5-phosphate and reduced nicotinamide adenine dinucleotide phosphate (NADPH) [23]. The role of NADPH is to provide the hydrogen atoms necessary for the formation of many molecules in chemical reactions (steroids, neurotransmitters, fatty acids, amino acids) [8]. NADPH is also involved in the synthesis of glutathione [8,23]. Glutathione is involved in the reduction of reactive H_2_O_2_ into H_2_O and is essential for the body’s defence response against oxidative stress [8]. Disruption of the conversion of H_2_O_2_ results in its conversion into hydroxyl free radicals. This leads to damage to neuronal cells, which can manifest as delirium [8].

### 1.2. Thiamine Deficiency in Critical Illness

**Figure 1:**
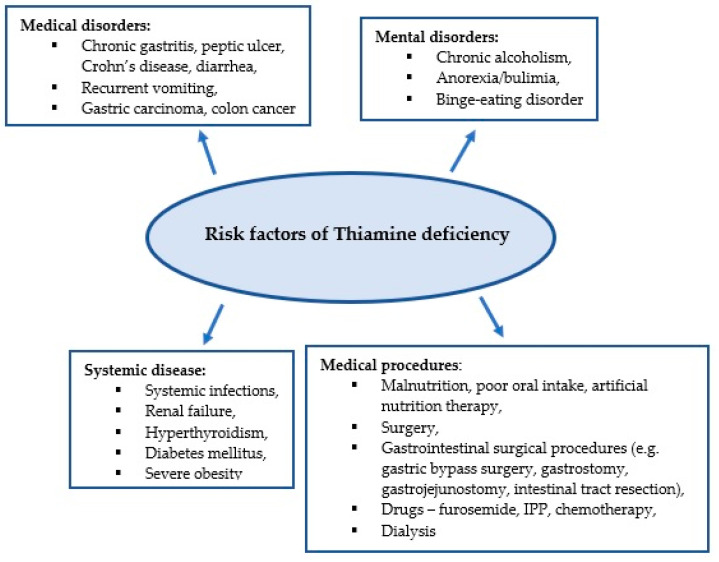
Risk factors for thiamine deficiency.

Thiamine plays an important role in energy and oxidative metabolism, and thiamine deficiency leads to impairment of these processes [23,24]. Thiamine stores in the body are low and thiamine deficiency can develop within 2–3 weeks of inadequate intake [5]. Due to the low specificity of symptoms associated with thiamine deficiency, its diagnosis is challenging for clinicians. It is estimated that approximately 20% of critically ill patients have thiamine deficiency confirmed by biochemical tests [6]. Medical conditions in critically ill patients can predispose to thiamine deficiency, which in turn contributes to poor response to the treatment administered, as well as the development of complications such as delirium, unexplained lactic acidosis, heart failure, gastrointestinal dysfunction, and critical neuropathy [25]. Figure 1 presents the predisposing factors for thiamine deficiency [7,8,22,26,27].

Conditions in which metabolic processes are activated and require increased metabolic demands associated with infection increase the risk of thiamine deficiency. It is estimated that between 10% and 70% of patients in septic shock are thiamine deficient [4,28]. Impaired oxygen delivery has been considered the main cause leading to multi-organ failure in septic shock. However, increasing attention is being paid to the role of mechanisms independent of oxygen supply, such as mitochondrial dysfunction, wherein thiamine is an essential cofactor [4].

Thiamine deficiency also occurs in conditions where the supply of thiamine from food is reduced, or its absorption is impaired. These include oral and gastrointestinal cancers and surgical procedures in the gastrointestinal tract [29,30].

The inability to take food orally due to critical illness leads to thiamine deficiency [25]. Paradoxically, inadequate industrial nutrition in the ICU may also contribute to the development of thiamine deficiency [7]. Due to the increased supply of simple carbohydrates, thiamine requirements are increased. In addition, the presence of electrolyte disturbances, including hypomagnesaemia, decreases thiamine pyrophosphate synthesis and thus contributes to thiamine deficiency [7,25,31].

In addition, some drugs used in the ICU may contribute to the development of thiamine deficiency [7]. Proton pump inhibitors (PPIs) reduce thiamine absorption, furosemide causes increased thiamine excretion. Decreased thiamine levels have also been reported in patients undergoing dialysis therapy [7,32,33,34].

The main aim of this study is to review the available literature and clinical trials that focus on the effect of thiamine supplementation to treat and prevent delirium in critically ill patients.

## 2. Methods

### 2.1. Study Design

A scoping review was conducted in the second quarter of 2021.

### 2.2. Definition of Scoping Review

Scoping reviews represent a relatively new approach to synthesizing evidence, and there is currently little guidance on deciding between a systematic review and a scoping approach during the synthesis of evidence, especially when the literature has not yet been comprehensively reviewed or shows a large, complex, or heterogeneous nature that cannot be subject to a more thorough systematic review [35].

### 2.3. Research Question

The following research question was formulated: Is routine thiamine supplementation associated with a reduced incidence of delirium among ICU patients?

### 2.4. Search Strategy

Based on the research question, the following inclusion criteria were established:

- Secondary studies (e.g., meta-analyses, systematic reviews, overviews, narrative reviews, integrative reviews) that summarized the state of research in the field and its gaps and provided directions for future research were included.
- Conceptual analyses used to summarize knowledge based on the literature review were included to provide a comprehensive map of the conceptual framework available in the field of research.
- Articles written in English and published in journals indexed in PubMed, Cochrane Library, Ovid, and ClinicalTrials.gov databases, until 30 June 2021.

Therefore, case reports, editorial and expert reports, as well as primary quantitative and qualitative research were excluded.

**Figure 2:**
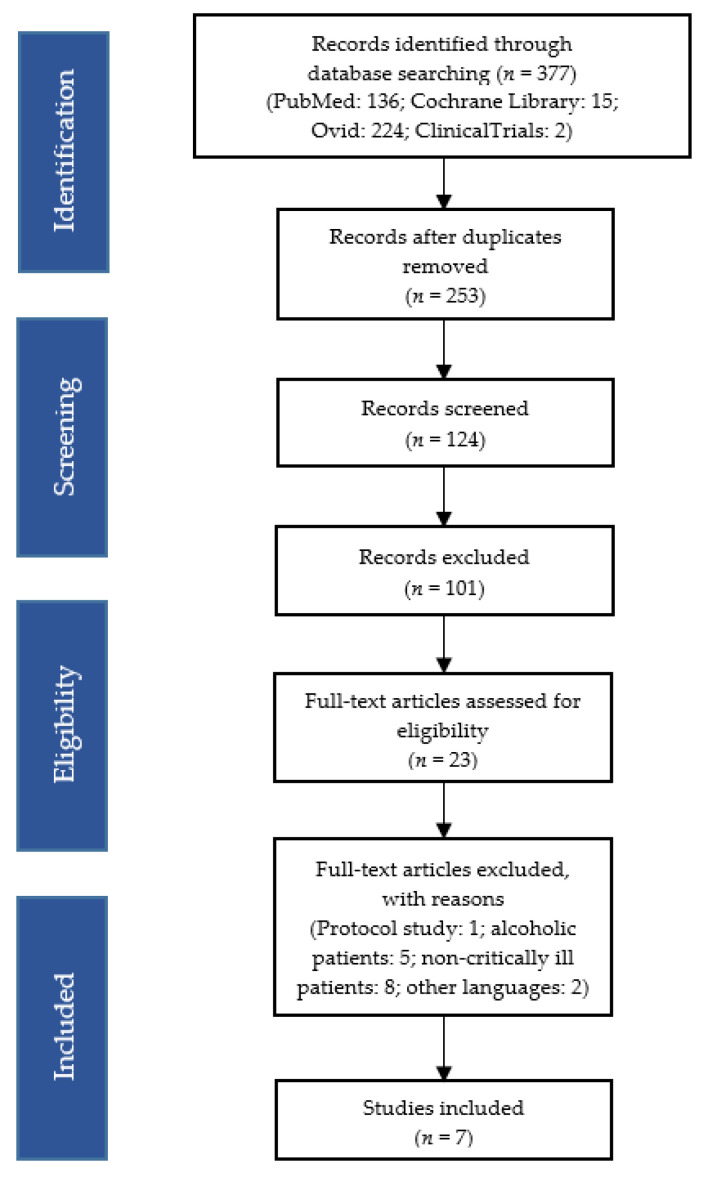
Flow diagram of study selection and inclusion.

The following terms, linked to the Boolean operator “AND/OR”, were used: “delirium”, “thiamine”, “thiamine supplementation”, “thiamine supplementation in critically ill patients”, “thiamine dosage in critically ill patients”. The compiled studies were checked independently by two reviewers who analysed their titles and abstracts for inclusion criteria. The full texts of eligible reviews were then retrieved and reviewed independently by the same two researchers. The list of literature cited in the included reviews was examined and their citations, documented in the Scopus database, were also checked. Decisions on the final inclusion of the 7 studies were based on reading the full text and agreement between the researchers (Figure 2).

### 2.5. Data Extraction

The included reviews and conceptual analyses were read carefully. The following data were then extracted: (a) objective(s); (b) study design (e.g., retrospective, observational, case-control); (c) target population; (d) conceptual framework, if available, used to design/explain the effectiveness of the intervention in the primary study included in the review; (e) interventions; (f) outcomes.

### 2.6. Analysis, Collation, and Summary of Data

Studies were evaluated using a formalized form of data extraction that included the following data: Clinical Trial ID, study design, aim, intervention, outcome measure, and result.

## 3. Results

One active clinical trial using thiamine as a preventive strategy has been identified in the ClinicalTrials.gov database. The clinical trial (NCT04214106) (Table 1) aims to evaluate the effectiveness of routine thiamine administration as an adjuvant in preventing delirium in patients admitted to the ICU. Thiamine supplementation has been routinely performed in the unit since 2016. The researchers want to compare the incidence of delirium in the era before and after the implementation of thiamine supplementation at a dose of 100–500 mg/day for at least one day. This study has been completed, but the results have not been published yet [36].

The randomised, double-blind, placebo-controlled, phase II study NCT02322892 (Table 2) used thiamine as an adjunctive therapy in patients undergoing cardiac surgery. The primary endpoint was lactate levels after surgery. Secondary outcomes were PDH activity, postoperative complications (incl. delirium), length of ICU and hospital stay, and mortality. In addition, the study assessed lactate levels 6 h after surgery, duration of mechanical ventilation, duration of vasopressors, cellular and global oxygen consumptions. Patients undergoing surgery were divided in a group receiving 200 mg of thiamine intravenously in 50 mL of 0.9% saline solution immediately before surgery and a placebo group given 50 mL of 0.9% saline solution, respectively. The patients received a second dose of thiamine or placebo after the surgery in the ICU. Thiamine administration showed no significant difference in the prevention of delirium. Delirium occurred in three (10%) patients in the thiamine group and in four (12%) in the placebo group. Other variables also showed no significant difference between the two groups. Only cellular and global oxygen consumptions were significantly higher in the thiamine group [37].

The clinical trial IRCT20190224042815N1 (Table 2) assessed the effect of thiamine on the prevention of postoperative delirium in patients undergoing gastrointestinal surgery admitted to the ICU. The most common reason for hospital admission was gastrointestinal cancer. A total of 96 patients were included in the study and were divided into a group receiving intravenous thiamine 200 mg/day and a group receiving intravenous 0.9% saline solution for three days. The validated Persian version of the CAM-ICU was used to assess delirium, and the level of sedation was measured using the RASS scale, every 12 h [38]. The outcome showed that the incidence of delirium was significantly lower in the thiamine group than in the placebo group. On the first day, delirium occurred in four (8.3%) patients receiving thiamine and in 12 (25%) patients receiving saline solution. On the following day, the number of patients with delirium in the experimental group decreased to two (4.2%) and in the placebo group to 10 (20.8%). On day 3, delirium occurred in three (6.2%) and eight (16.7%) patients in the thiamine and placebo groups, respectively. Secondary outcomes of the study: mean morphine equivalent dose and duration of mechanical ventilation were not statistically significantly different between groups [39].

Nakamura et al. (NCT03263442) (Table 2) assessed the effect of high-dose thiamine on preventing delirium in cancer patients undergoing allogeneic stem cell transplantation. Delirium was identified in 24% of patients enrolled in the study. In this randomised, double-blind, placebo-controlled study, a group of patients (*n* = 28) received 200 mg IV of thiamine three times a day for seven days, a control group (*n* = 33) received 100 mL of saline solution. The Delirium Rating Scale (DRS) was used to assess the incidence of delirium. Finally, there was no statistically significant difference in the incidence of delirium between the thiamine and placebo groups. Delirium was identified in seven (25%) in the experimental group and in seven (21%) in the control group. Additionally, serum thiamine levels were analysed in the study. Before the study, thiamine levels were not significantly different between the two groups. After surgery and seven daily high doses of thiamine supplementation, thiamine levels were significantly higher in the study group compared to the placebo group, 275 nmol/L vs. 73 nmol/L. In this study, thiamine levels were not found to be a risk factor for delirium. However, other potential factors were identified, such as the stage of cancer, corticosteroid intake, and nutritional status (nutritional markers: albumin, total protein, calcium, and bicarbonate levels) [40].

A prematurely ended clinical trial (NCT03509350) (Table 2) using thiamine in combination with vitamin C and hydrocortisone was identified. The main objective was to evaluate the effect of the therapy used on the number of ventilator- and vasopressor-free days in patients with sepsis. Thirty-day mortality in the study group was also assessed, as well as changes in sequential organ failure assessment score (SOFA) up to day 4, length of ICU stay, length of hospital stay, delirium/coma free days, and renal replacement therapy free days. In total, 501 patients were included in the study, of which 252 were allocated to the intervention group and 249 to the control group after randomisation. The intervention group received intravenous vitamin C 1.5 g, thiamine 100 mg, and hydrocortisone 50 mg every 6 h until 96 h, death, or discharge from the ICU. The control group was administered placebo, respectively. Both primary, secondary, and exploratory outcomes did not prove statistically significant between the two groups. In this study, the RASS and CAM-ICU scales were used to assess days free of coma and delirium. Days free from coma/delirium were assessed in 273 patients in the intervention group and in 241 in the control group. The median was four days in both groups (range 2–5 days) [41].

**Table 2:** Clinical trials.

Clinical Trials ID	Study Design	Interventions	Outcome Measure	Results
NCT02322892 [37]	Randomized, double-blind, placebo-controlled	Thiamine 200 mg IV (Experimental) vs. Normal saline IV (Placebo) before and after surgery	I: Postoperative lactate level. II: PDH activity, postoperative complications (incl. delirium), length of ICU and hospital stay, mortality Additional: lactate level (6 h after surgery), duration of mechanical ventilation, duration of vasopressors, cellular and global oxygen consumptions	There was no difference in clinical outcomes between the group of patients receiving thiamine and placebo. Postoperative cellular and global consumption were significantly higher in patients receiving thiamine.
IRCT20190224042815N1 [39]	Randomized, double-blind, placebo-controlled	Thiamine 200 mg IV (Experimental) daily vs. Normal saline IV (Placebo) for three days	I: Incidence of postoperative delirium II: Average morphine equivalent dose and duration of mechanical ventilation	The incidence of delirium was significantly lower in the thiamine group than in the placebo group.
NCT03263442 [40]	Randomized, double-blind placebo-controlled	Thiamine 200 mg IV (Experimental) three times daily for seven days vs. Normal saline IV three times daily for seven days (Placebo)	I: Incidence of delirium after allogeneic HSCT II: Post-HSCT blood thiamine levels, other potential risk factors for delirium	Thiamine use did not prevent delirium. Other potential risk factors: cancer progression, corticosteroid exposure, nutritional status.
NCT03509350 [41]	Multicentre, randomized, double-blind, adaptive-sample-size, placebo-controlled	Vitamin C 1.5 g IV, Thiamine, 100 mg IV, hydrocortisone 50 mg IV every 6 h vs. placebo for 96 h or until discharge from the intensive care unit or death.	I: number of consecutive ventilator- and vasopressor-free days in the first 30 days following the day of randomization II: 30-day mortality Exploratory Outcomes: mortality before ICU discharge, Mortality at 180 d, change in SOFA score, length of ICU stay, length of hospital stay, coma-/delirium-free days, kidney replacement therapy–free days	No statistically differences between groups

IV, intravenous; ICU, intensive care unit; PDH, pyruvate dehydrogenase; HSCT, hematopoietic stem cell transplantation.

Table 3 presents the main aims and results of two retrospective studies. Park et al. conducted a single-centre observational study among patients in septic shock. The primary outcome of the study was delirium-free days. Subsequently, the frequency of delirium over 14 days, delirium-coma-free days, duration of delirium, duration of hospital and ICU stay, and 28-day mortality were observed. Patients who fulfilled the inclusion criteria were given intravenous vitamin C 3 g every 12 h or 1.5 g every 6 h and thiamine 200 mg every 12 h. The median duration of therapy was 2 days (1–4 days). The study population was divided into treatment and control groups. The Richmond Agitation Sedation Scale (RASS) was used to measure sedation assessment and the validated Korean version of the Confusion Assessment Method for ICU (CAM-ICU) was used to assess delirium. Assessment was performed at least once per nursing shift. Other data were extracted from the registry and electronic medical records. Median delirium-free days were not significantly different between the treatment and control groups and were 11 days (5–14) vs. 12 days (6–14), respectively. Secondary outcomes were also not significantly different between the two groups. However, this study has a significant risk of bias, which may affect the results. First, the duration of therapy was short, with a median of two days. Secondly, the dose of vitamin C and thiamine may have been insufficient, given that patients in shock are particularly at risk of developing a deficiency of these vitamins. Therefore, it was also a limitation of this study that serum vitamin C and thiamine levels were not measured in patients. Fourthly, this study was not randomised, and the decision to start vitamin therapy and to stop it was made by the physician [42]. Among cancer patients, thiamine deficiency may cause delirium [43]. Onishi et al. conducted a retrospective study among cancer patients diagnosed with delirium using the Diagnostic and Statistical Manual of Mental Disorders 5th edition (DSM-V). Of the 71 patients included in the study, 45.1% had thiamine deficiency. All these patients were given intravenous thiamine. Improvement was observed in 59.3% of patients. Finally, a statistically significant association was found only between chemotherapy and thiamine deficiency. Other variables such as age, gender, albumin level, BMI, lactate dehydrogenase (LDH) level, gastrointestinal malignancy, performance status, hormonal therapy, radiotherapy, surgery, diabetes, hypertension, and cardiac disorders were not statistically significant [30].

## 4. Discussion

Delirium is a common disorder that occurs in critically ill patients in the ICU. It affects outcomes, treatment costs, and the occurrence of long-term cognitive impairment [44]. Among post-surgical patients, the prevalence of delirium is 12.8–36.8% and increases with age [45,46]. In cancer patients, the rate is estimated to be 22–44% [47]. In a retrospective study by Sonneville et al., 53% of patients with sepsis had delirium [48]. In a study by Ely et al. delirium occurred in up to 81.7% (183/224) of ICU patients [17]. In the same study, the six-month mortality rate was significantly higher in patients who developed delirium (34% vs. 15%). In addition, patients with delirium spent 10 days longer in the unit. Additionally, patients with delirium were diagnosed with a higher incidence of cognitive impairment at hospital discharge [17]. In a study by Pandharipande et al. in which 821 patients from ICUs (medical and surgical) for respiratory failure, cardiogenic or septic shock were included, 74% developed delirium. Longer duration of delirium was an independent risk factor for poorer global cognition and executive function scores, at three and 12 months after discharge [49].

Delirium has an adverse effect on patient outcomes, prolongs the length of stay in the ward, increases mortality, and causes cognitive and functional impairment in patients [17,49,50]. Therefore, the most effective method to minimise adverse effects would be prevention [51]. Furthermore, a comprehensive review by Friedman et al. showed that delirium medications are more effective in preventing than in treating delirium [52]. Haloperidol and second-generation antipsychotics have shown significant effects in preventing postoperative delirium. In patients undergoing hip surgery, iliac fascia block with bupivacaine reduced the incidence of delirium. Gabapentin administered perioperatively to patients undergoing spinal surgery had a positive effect. The use of continuous intravenous infusion of dexmedetomidine to induce sedation in mechanically ventilated patients in the ICU showed a lower rate of delirium than medications, such as midazolam, propofol, and morphine. Among intraoperative interventions to prevent delirium, maintaining a lower level of sedation with propofol and using a single dose of ketamine for induction of anaesthesia were effective. Interestingly, both haloperidol and second-generation antipsychotics were not effective in treating ongoing delirium [52].

In recent years, there has been a return of interest in vitamin therapy in critically ill patients [1]. The effect of vitamin D3 supplementation on improving outcomes among ICU patients has been studied [53,54]. Thiamine administered at 200 mg IV for seven days contributed to lower lactate levels and lower mortality in septic shock patients [2]. Some researchers have pointed out that the occurrence of delirium may be related to thiamine deficiency [8]. Thiamine is an essential cofactor for the enzymes PDH and α-KGDH, which are required for glucose metabolism. It plays an important role in the Krebs cycle, and the pentose phosphate pathway. It is involved in the production of ATP as well as NADPH [55]. Due to its water-soluble nature, thiamine cannot be stored. It should therefore be delivered to the organism through appropriate supplementation and continuous therapeutic adjustments [25].

A state of increased metabolism, industrial nutrition without adequate micronutrient enrichment, some drugs used in the ICU, and medical procedures predispose to thiamine deficiency among critically ill patients [5]. Thiamine deficiency in shock patients is estimated to be 20–70% [28,56]. In a study by Onishi H et al., 45.1% of cancer patients were thiamine deficient [30]. In gastrectomy patients, thiamine deficiency was diagnosed in 25.7% [57]. In a study conducted in an ICU, thiamine deficiency was found among 11–70% [5]. Considering the low risk of side effects and the low cost, thiamine could prove to be a very good alternative to other drugs used to prevent delirium.

In the clinical trials identified by us, only the IRCT20190224042815N1 trial showed a beneficial effect of thiamine use as a preventive agent for delirium among patients undergoing gastrointestinal surgery in a critical condition [39]. Other studies in which this was a primary or secondary outcome showed no significant statistical difference between the thiamine (alone or in combination) and placebo groups [37,40,41]. The clinical trial NCT04214106, comparing the incidence of delirium before and after the implementation of routine thiamine administration to ICU patients, has been completed but the results have not been published yet [36]. In a retrospective study by Onishi et al., thiamine deficiency was identified in 45.1% of cancer patients. After intravenous thiamine administration, improvement was observed in almost 60% of patients. This study focused on cases of cancer patients referred for psychiatric consultation, so the findings cannot be generalised. Furthermore, not all patients had their serum thiamine levels tested. Additionally, this study did not identify patients with alcohol dependence, which may have influenced the results [30].

Due to the very small number of studies, as well as the high heterogeneity, it is not possible to compare results and draw clear conclusions from the review. Firstly, there is no standard dosing schema for thiamine. Andersen et al. administered thiamine before and after surgery, Moslemi et al. used a dose of 200 mg once daily for three days, while Nakamura et al. applied 200 mg thiamine three times daily for a duration of seven days [39,40]. In the study by Sevransky et al., a dose of 100 mg thiamine was administered in combination with vitamin C and hydrocortisone [41]. The studies differed not only in dose, but also in timing of supplementation and frequency of administration. The frequency and methods of delirium assessment also differed between studies. For example, Moslemi et al. assessed sedation using the RASS scale and delirium by the CAM-ICU scale every 12 h. Meanwhile, Nakamura et al. assessed using the 10-item DRS scale three times a week, and if positive, the frequency was increased [39]. Onishi et al. used the Diagnostic and Statistical Manual of Mental Disorders 5th edition (DSM-V) as a screening test for delirium [30]. A high heterogeneity also existed in the patient population. The studies included patients undergoing various surgeries (cardiac surgeries, gastrointestinal surgeries, hematopoietic stem cell transplantation (HSCT)), patients in septic shock and with cancer [30,37,39,40,41]. Furthermore, not all studies measured thiamine levels prior to intervention. Considering that critically ill patients are at risk of thiamine deficiency, doses may have been insufficient. In the study by Andersen et al., preoperatively, thiamine levels were comparable in both groups. Immediately and 6 h after surgery, thiamine levels were significantly higher in the group receiving intravenous thiamine than placebo (1200 nmol/L vs. 9 nmol/L and 1200 nmol/L vs. 10 nmol/L). In addition, after treatment, no patient in the experimental group had thiamine deficiency. In the placebo group, thiamine deficiency occurred in three patients immediately after surgery and in five patients 6 h after surgery [37]. Similarly, in the study by Nakamura et al. before surgery, the mean thiamine score was comparable in both groups. However, after seven days of supplementation, the level was significantly higher in this group. In the thiamine group, significantly higher levels persisted until day 22 after transplantation. During hospitalisation, thiamine deficiency was identified in 39% of patients in the thiamine group and 64% in the placebo group. The frequency of deficiency at day 8 was significantly higher in the placebo group (53% vs. 0%) and persisted until day 22 after surgery (55% vs. 13%) [40].

It should also be noted that, in most studies, the sample size was small, and the intervention time was short. A large multicentre study by Sevransky et al. that registered 501 patients had to be terminated prematurely for administrative reasons, which may have affected later results [41]. We also noted that no study, even those where delirium was the primary outcome, differentiated the type of delirium.

Among patients in the ICU, the most common type is hypoactive (apathy, lethargy, withdrawal, impaired response to stimuli) and mixed (transition of one state to another). It is estimated that less than 5% of ICU patients develop the hyperactive form of delirium (agitation, anxiety). Alack of regular screening may lead to undiagnosed delirium [16,58,59].

## 5. Limitations

There is a lack of information in the literature regarding thiamine dosing standards. Thiamine levels before implementing treatment are often not performed. Due to the small number of studies, no clear conclusions can be made about how and with what results thiamine treatment can be implemented. The centres that have undertaken trials have often not differentiated between the type of delirium experienced by the patients included in the study. Few centres use instruments to assess delirium already at the screening stage, which could benefit delirium prevention. In most publications, studies were conducted on a small group of ICU patients.

## 6. Conclusions

The small number of studies and heterogeneity make it impossible to draw conclusions confirming the use of thiamine as an adjuvant in the prevention and treatment of delirium in critically ill patients. There is a need for large-scale, high-quality randomised clinical trials to confirm the beneficial effects of thiamine in the prevention and treatment of delirium.

## 7. Implications for Practice

Because delirium has a multifactorial etiology, it is important to consider that thiamine may not have beneficial effects in all critically ill patients. Thiamine therapy did not prove to be insignificant in the group of patients after abdominal surgery. The implementation of thiamine treatment may have benefits for ICU patients after abdominal surgery. The selection of appropriate thiamine doses should be made after careful case-by-case analysis. Comparing before and after administration of thiamine levels, the assessment of delirium on both groups, may contribute to a scheme that can be successfully adopted and used in the ICU departments. However, it is important to note that it is necessary to implement the scheme with appropriate instruments for the assessment of delirium. With the knowledge of delirium assessment methods, further studies on the use of thiamine may provide results that will benefit the treatment of patients in the ICU with delirium.

## Figures and Tables

**Table 1:** Active trial.

Clinical Trial ID	Study Design	Aim
NCT04214106 [36] *	Retrospective, Observational, Case-Control	Investigate whether the routine use of thiamine has been associated with decreased prevalence of delirium among ICU patients when compared to the pre-routine thiamine administration era.

* No results posted.

**Table 3:** Retrospective study.

First Author, Year	Aim	Results
Park JE et al. (2020) [42]	Evaluate the impact of early combination therapy with vitamin C and thiamine on ICU delirium-free days in patients with septic shock.	1. Vitamin C and thiamine therapy did not increase the number of delirium-free days in septic shock patients. 2. Therapy also had no effect on other clinical outcomes (number of coma-free days, incidence of delirium, duration of delirium, length of stay in hospital, ICU, and 28-day mortality).
Onishi H et al. (2021) [30]	Percentage of patients with TD and characteristics of cancer patients who developed delirium	1. Thiamine deficiency was found in 45% of patients with delirium. 2. TD was associated with chemotherapy.

TD, thiamine deficiency.

## Data Availability

The authors declare that the data of this research are available from the correspondence author on request.
